# Association between presence of latrine and unclean child face on the prevalence of active trachoma among children aged 1 to 9 years in low-income setting: A systematic review and meta-analysis

**DOI:** 10.1371/journal.pone.0330077

**Published:** 2025-08-07

**Authors:** Leykun Berhanu, Chala Daba, Belay Desye, Abebe Kassa Geto, Gete Berihun

**Affiliations:** 1 Department of Environmental Health, College of Medicine and Health Sciences, Wollo University, Dessie, Ethiopia; 2 Department of Public Health, College of Health Sciences, Woldia University, Woldia, Ethiopia; 3 Department of Environmental Health, College of Medicine and Health Sciences, Debre Markos University, Debre Markos, Ethiopia; Mizan-Tepi University, ETHIOPIA

## Abstract

**Background:**

Trachoma is the leading infectious cause of blindness worldwide, primarily affecting populations in low-income countries with poor sanitation and hygiene conditions. The World Health Organization has set a goal to eliminate trachoma as a public health problem. However, progress towards this goal has been uneven across different regions. This systematic review and meta-analysis aimed to synthesize the available evidence on the association between the presence of latrine facilities and unclean child faces with the prevalence of active trachoma among children aged 1 to 9 years in low-income country settings. The findings from this study can help guide the design of more targeted interventions to reduce the burden of trachoma in vulnerable populations.

**Materials and methods:**

A total of 2695 articles were searched from PubMed, Hinari, African Online Journals, Google, Google Scholar, Science Direct, and Semantic Scholar and exported to STATA version 17 for analysis. The levels of heterogeneity among studies were assessed using I2 and p-values. The findings of the meta-analysis were presented using a table, graph, and forest plot with a 95% confidence interval. A P-value of less than 0.05 was considered statistically significant.

**Result:**

Among 2695 articles searched, 16 of them were selected for meta-analysis. The pooled prevalence of active trachoma was 21.10 (95% CI; 14.18, 28.02). The finding indicated high heterogeneity among the included studies (I^2^ =99.3%, p<0.0001). There was a statistically significant association between the presence of a latrine (POR=0.10, 95% CI: 0.00, 0.19) and unclean child face (POR=1.30, 95% CI: 1.08, 1.53) and the pooled prevalence of active trachoma among children aged 1 to 9 years in low-income countries.

**Conclusion:**

The present study revealed that the pooled prevalence of active trachoma was high as compared to the WHO trachoma eradication goal. The presence of latrine facility and unclean child face were significantly associated with the prevalence of active trachoma among children aged 1 to 9 years in low-income countries. Hence it is recommended to improve access to latrines, promote child facial hygiene, and intensify overall trachoma control in low-income countries.

## Background

Trachoma is the most common infectious cause of blindness globally, impacting people in low-income nations where poverty and limited access to resources for health, sanitation, and clean water are persistent issues [1,2]. The bacteria known as Chlamydia trachomatis is the cause of trachoma. It can spread by close quarters, direct touch, and the eye-seeking fly Musca Sorbens, which deposits its eggs on human refuse [1]. According to the World Health Organization (WHO), trachomatous inflammation follicular (TF) and trachomatous inflammation (TI) are a sign of active trachoma, usually associated with conjunctiva C. trachomatis infection [3]. WHO grades TF and or TI are associated with active trachoma. Based on the frequency of TF, trachoma endemicity can be non-endemic (less than 5%), hypo-endemic (between 5 and 10%), mesoendemic (between 10 and 30%), and hyper-endemic (more than 30%) [4].

According to estimates from the WHO, trachoma causes 3.6% of all cases of blindness and is endemic in 56 countries, the majority of which are in Africa and the Middle East [5]. Africa, the Middle East, Central and South America, Asia, Australia, and Africa are the 42 nations where active trachoma is endemic. Of these, 200 million people live at risk, approximately 150 million have active trachoma, with 6 million becoming blind as a result of the disease’s devastating complications [6]. Studies have indicated that the highest risk groups for trachoma infection and transmission are mothers and young children, particularly those under the age of nine [7].

Previous studies have identified several key risk factors commonly observed in children, including infrequent face washing, high populations of food-seeking flies, overcrowding, insufficient latrines, the presence of livestock in living areas, lack of proper chimney ventilation, and inadequate garbage disposal facilities. Addressing these factors is crucial for improving child health outcomes [1,8]. Among these factors, the lack of latrine facilities plays an important role. Inadequate sanitation can lead to increased exposure to pathogens, significantly impacting children’s health and well-being. Addressing this issue is essential for reducing disease transmission and improving overall hygiene [1]. Consequently, the World Health Organization has mandated that blinding trachoma be eradicated by 2020 [9]. Donating Zithromax (azithromycin) has allowed for the extension of efforts to reduce the illness burden associated with these goals. Annual treatments increased dramatically from 1 million doses in 1998 to 47.8 million in 2012 [10] with more than 30 countries launching national trachoma strategies and elimination initiatives [11]. However, these efforts alone will not lead to the sustainable elimination of blinding trachoma, and it is widely recognized that a scale-up of the full SAFE (Surgery, Antibiotics, Facial cleanliness, Environmental Improvement) strategy is needed to reach 2020 targets [12]. For example, no single instrument can be proposed for the F and E components of SAFE; instead, sustained reductions in disease burden require improvements in environmental circumstances, most notably in hygiene and sanitation practices such as construction and appropriate utilization of latrine facilities and hand and face hygiene promotion are an important strategy to reduce the burden of trachoma [9,13].

Numerous studies demonstrate a direct correlation between the availability of latrine facilities and a reduced risk of active trachoma. A 2015 meta-analysis and systematic review looked at 33 publications from 13 different countries. The results showed that access to a toilet was associated with a 30% lower probability of active trachoma versus no access at all [9]. The Musca Sorbens housefly, which breeds in human waste, is the primary vector of trachoma transmission. The presence of latrine facilities reduces feces, thereby eliminating a major breeding ground for disease-carrying flies. When there are fewer flies, the trachoma-causing bacteria, Chlamydia trachomatis, has a lower chance of mechanically spreading. In addition to latrines, access to clean water is another vital component of improved sanitary infrastructure. If homes have access to clean water and toilets, they can take better care of the environment and themselves. By doing this, the likelihood of the Chlamydia bacteria spreading through direct contact, sharing clothing or towels, and contaminated hands or faces is decreased. It is often assumed that having access to a latrine indicates a higher social status. For homes having latrines, access to extra healthcare services and health education may also be enhanced. This could lead to improved general health behaviors and outcomes, such as a lower incidence of trachoma [14].

Increased eye and nasal discharge, which may contain significant concentrations of the Chlamydia trachomatis bacterium, is a common symptom of active trachoma in children. Through direct contact or contaminated hands or towels, the discharge can quickly spread the infection to other kids if their faces are not cleansed correctly. The primary vector for trachoma transmission is the Musca sorbens housefly, which is attracted to the nasal discharge and eyes of unclean children’s faces. These flies can transfer bacteria from one child’s face to another’s eyes, facilitating the spread of the infection. One of the main factors contributing to the spread of trachoma is the mechanical transfer by the fly vector. Youngsters who have dirty faces are more prone to contract trachoma again and again. Over time, more severe illness and consequences of corneal scarring and blindness might result from recurrent infections. Since they frequently have the greatest rates of active infection, children are the primary reservoir for Chlamydia trachomatis. Children with unclean faces provide a continual source of bacteria that can spread to other people in the community. Therefore, a key element of the SAFE approach for trachoma elimination is interventions that encourage facial cleanliness in children, such as face-washing programs. Keeping children’s faces clean reduces the bacterial load, prevents flies from spreading the infection, and diminishes the community’s reservoir of disease [15–18].

Maintaining facial cleanliness is crucial for significantly reducing the incidence of trachoma. Researchers found that children who skipped face washing had a 4.2-fold higher risk of active trachoma. As the frequency of face washing decreased and the children continued to have un-cleaned faces free of discharge, the likelihood of active trachoma increased [19]. Other researchers have provided the greatest evidence of the link between trachoma and hygiene factors [9,15,18,20–23].

The absence of a comprehensive systematic review and meta-analysis on this topic is identified as a significant gap in the proposed study which focuses on the association between latrine availability, facial cleanliness, and the prevalence of active trachoma in children aged 1to 9 years in low-income countries. Since trachoma rates are higher in low-income countries, those will be the focus of the study. More contextually relevant data may be generated to help guide actions in the most afflicted areas by restricting the geographic scope to particular contexts. The age range of 1 to 9 years will be the focus of the study, as this is the established age range where trachoma risk is highest in children. Focusing on this target group will yield insights that can tailor preventative and control measures for the most vulnerable population. Another identified gap is the presence of inconsistent findings from primary studies regarding the association between latrine access and the prevalence of active trachoma. The study’s meta-analysis component seeks to measure the magnitude of the association between the two main variables (the presence of latrines and unclean faces) and the prevalence of trachoma. Prioritizing and directing the execution of the most successful interventions can be aided by this. The study aims to provide a robust and current understanding of the relationship between these factors to supporting public health actions and policies aimed at reducing trachoma prevalence in resource-limited settings.

The proposed study intends to close the above-mentioned gaps by carrying out a comprehensive systematic review of the literature on the topic and a meta-analysis to measure the degree of correlation between dirty child faces, the presence of latrines, and the prevalence of active trachoma in the target population of children living in low-income environments.

## Materials and methods

### Protocol registration

To prevent redundant work, the relevance of the topic is verified using key databases, such as Embase, Cochrane Library, PROSPERO, and Episimonikos. A protocol was developed, submitted, and registered with the International Prospective Register of Systematic Reviews (PROSPERO) on June 8, 2024, under the registration number CRD42024573742.

### Search strategy

Relevant articles were identified by searching electronic databases, including PubMed, Science Direct, Hinari, African Online Journal, Semantic Scholar, and Google Scholar. A search strategy was developed using Boolean operators “AND” and “OR,” with synonyms for each search term employed to broaden the literature search. Additionally, filters were applied to refine results based on criteria such as language, study design, and population characteristics. To avoid publication bias, gray literature was also searched on Google and through searching reference lists of the already identified articles. The articles obtained from each database were registered on each search day. This systematic review and meta-analysis study was conducted following the updated Preferred Reporting Items for Systematic Review and Meta-analysis (PRISMA) guideline (S1 Table**).**

### Eligibility criteria

#### Inclusion criteria.

**Study Design:** All observational studies (cross-sectional, case-control, or cohort studies) reporting the association between latrine presence and/or unclean child face with the prevalence of active trachoma were included.

**Population:** Children aged 1 to 9 years living in low-income countries, as defined by the World Bank classification were included in this systematic review and meta-analysis

**Outcome:** Prevalence of active trachoma, as diagnosed by clinical examination or grading of trachomatous inflammation-follicular (TF) and/or trachomatous inflammation-intense (TI) according to the WHO simplified trachoma grading system.

**Language:** Studies published in English.

#### Exclusion criteria.

**Study Design:** Randomized controlled trials, case reports, reviews, books, book sections, and commentaries were not included.

**Population:** Studies conducted in low-income countries, as defined by the World Bank classification.

**Intervention/Exposure:** Studies that did not assess the presence of latrine/toilet facility or unclean child face.

**Outcome:** Studies that did not report on the prevalence of active trachoma and its association with the presence of latrine and unclean child face were excluded.

**Language:** Studies published in languages other than English were also excluded.

**Insufficient data:** Studies that did not provide sufficient data to estimate the association between exposure and outcome of interest.

### Outcome assessment

The primary goal of this study was to determine the association between the presence of latrine and unclean child faces on the pooled prevalence of active trachoma among children aged 1 to 9 years in low-income countries. The number of research participants with active trachoma divided by the actual sample size was used to estimate this, which was then multiplied by 100. Furthermore, a pooled odds ratio was calculated to determine the association between the presence of a restroom and an unclean child’s face on active trachoma. Consequently, a systematic review and meta-analysis were done.

#### Operational definition.

**Presence of active trachoma:** The presence of trachomatous inflammation- follicular (TF) or trachomatous inflammation intense (TI) trachoma is observed in the child’s face [24].

**TF**: the occurrence of five or more follicles greater than 0.5 mm in diameter in the central part of the upper tarsal conjunctiva [25]

**TI:** is marked inflammatory thickening of the tarsal conjunctiva that obscures more than half of the normal deep tarsal vessels (25)

**No active trachoma**: Refers to the absence of TF and TI [26]

**Clean child face**: The absence of nasal or ocular discharge, dirt, or a fly on the child’s face [6,27,28].

**Unclean child face:** refers to the presence of discharge on the eyes, nose and/or fly on the face [29].

**Presence of latrine**: the availability of a latrine facility to be used by the family members and or the child.

### Study selection process

**Fig 1:**
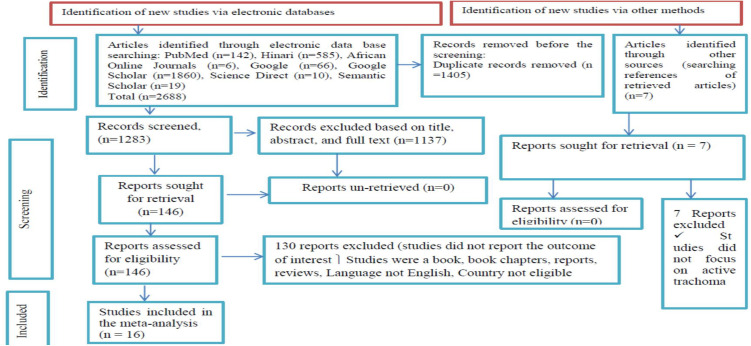
PRISMA flow diagram showing the selection of studies for systematic review and meta-analysis on the association between the presence of latrine and unclean child face on the prevalence of active trachoma among children aged 1 to 9 years in low-income countries, 2024.

Two reviewers, LB and AKG, independently evaluated studies based on their titles, abstracts, and full-text accessibility. The screened articles were compiled by LB and AKG to prepare them for further screening and selection. To resolve the difference between the two investigators, the study team members consulted additional reviewers, identified as GB. The study selection process has been simplified using the 2020 PRISMA flow diagram (Fig 1**).**

### Data extraction

After scanning relevant literature in the proposed databases and additional sources, all papers were exported to Endnote version 20 software. The amount of duplicate articles deleted from each database was tracked. After deleting all duplicates, the studies were assessed based on titles and abstracts against predetermined study inclusion criteria. A data extraction template was created, which included the author’s name, year of publication, sample size, country, continent, type of study design, response rate, active trachoma prevalence, potential risk factors for active trachoma prevalence, adjusted odds ratio, and confidence interval for each of the predictors were noted.

### Study quality and bias assessment

The Joanna Briggs Institute (JBI) quality rating instrument was used to assess the quality of papers for consideration in the review. LB, AKG, and GB independently perform study quality assessments using JBI criteria for a cross-sectional study. The criteria measured each of the articles out of 100 percent. After a thorough review, studies with a score of 50% and above for each study were identified as having low risk. Any disagreement in the quality assessment process was resolved by taking the mean score of the entire quality assessor. Studies with a quality evaluation indicator score of 50% or above [30] were classified as low risk (S2 Table**).** Egger’s and Begg’s tests were used to objectively determine publication bias. A funnel plot was also created to subjectively assess the presence of publication bias in articles included in this review.

### Data analysis and presentation

The data was extracted using an Excel sheet and exported to STATA version 17 software for analysis. The presence and level of heterogeneity among the selected studies were assessed using I^2^ and p-values. The heterogeneity of the studies was considered high risk (I^2^ = 98.9 p < 0.001). Due to the presence of high heterogeneity among the included articles, a random effect Meta-regression analysis model was used to measure the pooled prevalence and associated factors of active trachoma among children aged 1 to 9 years in low-income countries. Sensitivity analysis was performed to determine a single study effect on the pooled prevalence of active trachoma. The finding of the meta-analysis was presented using a table, graph, and forest plot with a 95% confidence interval. A p-value of less than 0.05 was considered statistically significant.

## Result

### Description of the included studies

Of the 16 articles included in the meta-analysis, 13 of them were published in Ethiopia [1,6,25–29,31–36] and one study each from Sudan [37], Gambia [38], and Uganda [39] In 16 articles, 9914 study participants were examined for the presence of active trachoma and 1998 of them were positive. The highest prevalence of active trachoma was reported to be 37.9% [32] and the lowest was 1.3% [39]. The highest JBI score was 87.5% [26] and the lowest was 50 [29,35,38,39]. (Table 1)

**Table 1:** Characteristics of the study included studying the prevalence of active trachoma and associated factors among children aged 1 to 9 years in low-income countries, 2024.

Author Year	Country	Continent	Study design	Diseased	Prevalence of active trachoma	Sample size	JBI score (100%)
Abdilwohab and Abebo, (2020) [31]	Ethiopia	Africa	Cross-sectional	148	17.8	831	62.5
Alambo et al (2020) [32]	Ethiopia	Africa	Cross-sectional	222	37.9	586	75
Alemayehu et al (2015) [33]	Ethiopia	Africa	Cross-sectional	105	15.6	671	62.5
Alkhidir and Elhag, (2018) [37]	Sudan	Africa	Cross-sectional	99	11	900	75
Ashine et al (2024) [27]	Ethiopia	Africa	Cross-sectional	116	15.8	736	75
Asres et al (2016) [28]	Ethiopia	Africa	Cross-sectional	71	12.1	586	75
Kedir et al (2021) [25]	Ethiopia	Africa	Cross-sectional	165	29.4	561	62.5
Mekonnen et al (2022) [34]	Ethiopia	Africa	Cross-sectional	39	21.91	178	62.5
Mengistu et al (2016) [35]	Ethiopia	Africa	Cross-sectional	224	36.7	611	50
Mohamed et al (2019) [29]	Ethiopia	Africa	Cross-sectional	35	4.3	823	50
Quicke et al (2013) [38]	Gambia	Africa	Cross-sectional	25	3.8	652	50
Reda et al (2020) [40]	Ethiopia	Africa	Cross-sectional	108	21.5	502	75
Tuke et al (2023) [36]	Ethiopia	Africa	Cross-sectional	157	29.2	538	75
Ndikuno et al(2022) [39]	Uganda	Africa	Cross-sectional	6	1.3	472	50
Yeshitila M et al (2022) [26]	Ethiopia	Africa	Cross-sectional	205	27	760	87.5
Golovaty et al, 2009 [1]	Ethiopia	Africa	Cross-sectional	273	53.9	507	87.5

### Subgroup analysis

**Fig 2:**
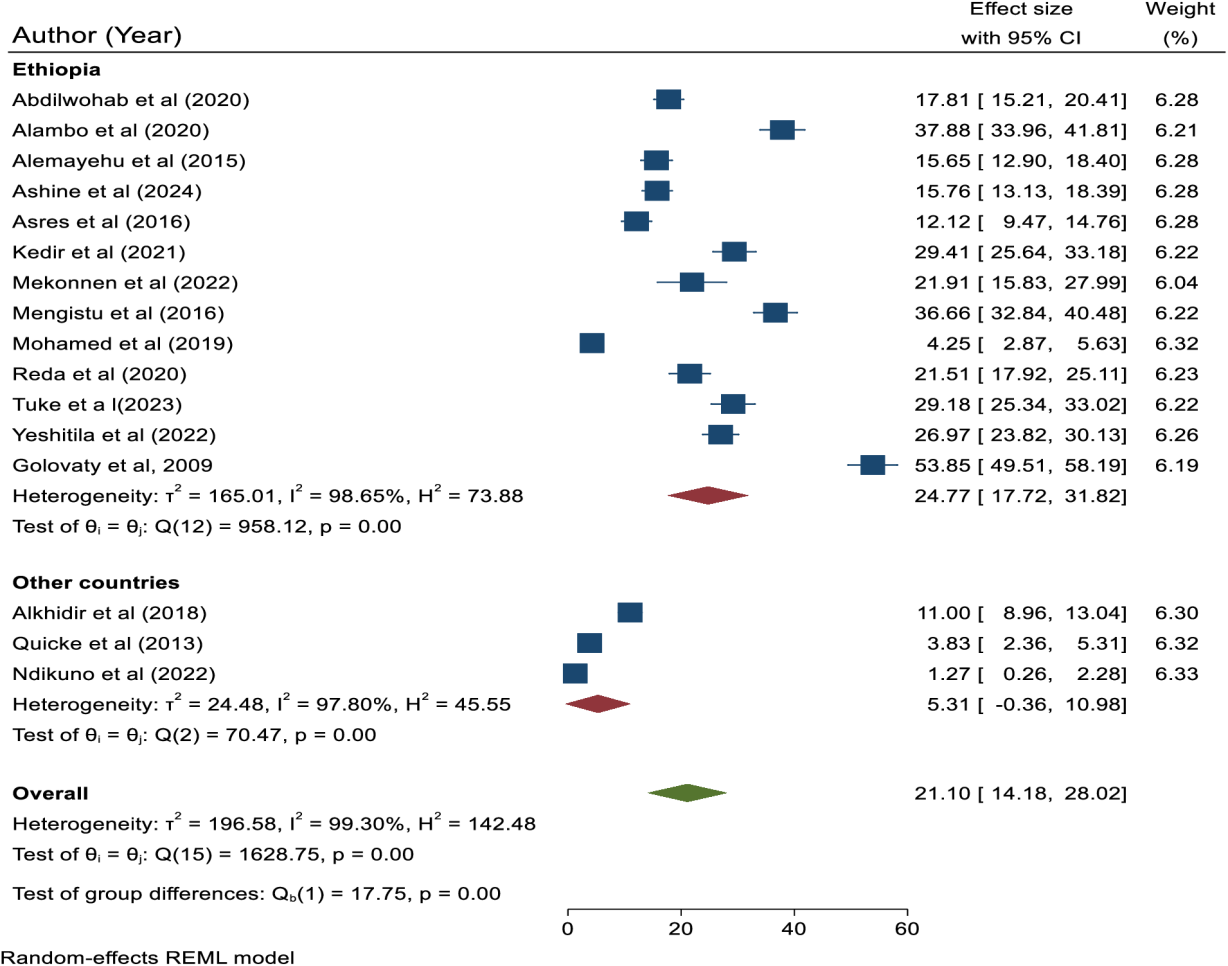
Subgroup analysis based on country category.

Subgroup analysis was done based on country category, publication year, and sample size. The subgroup analysis based on the country category showed that the highest prevalence of active trachoma was reported in Ethiopia with a prevalence of 24.77% (95% CI, 17.72, 31.82), while the lowest prevalence was reported in other countries where the prevalence of active trachoma was reported to be 5.31% (95%, CI: −0.36, 10.98). Moreover, the difference in the pooled prevalence of active trachoma among studies done in Ethiopia and other countries was statistically significant (p < 0.0001) (Fig 2**).**

**Fig 3:**
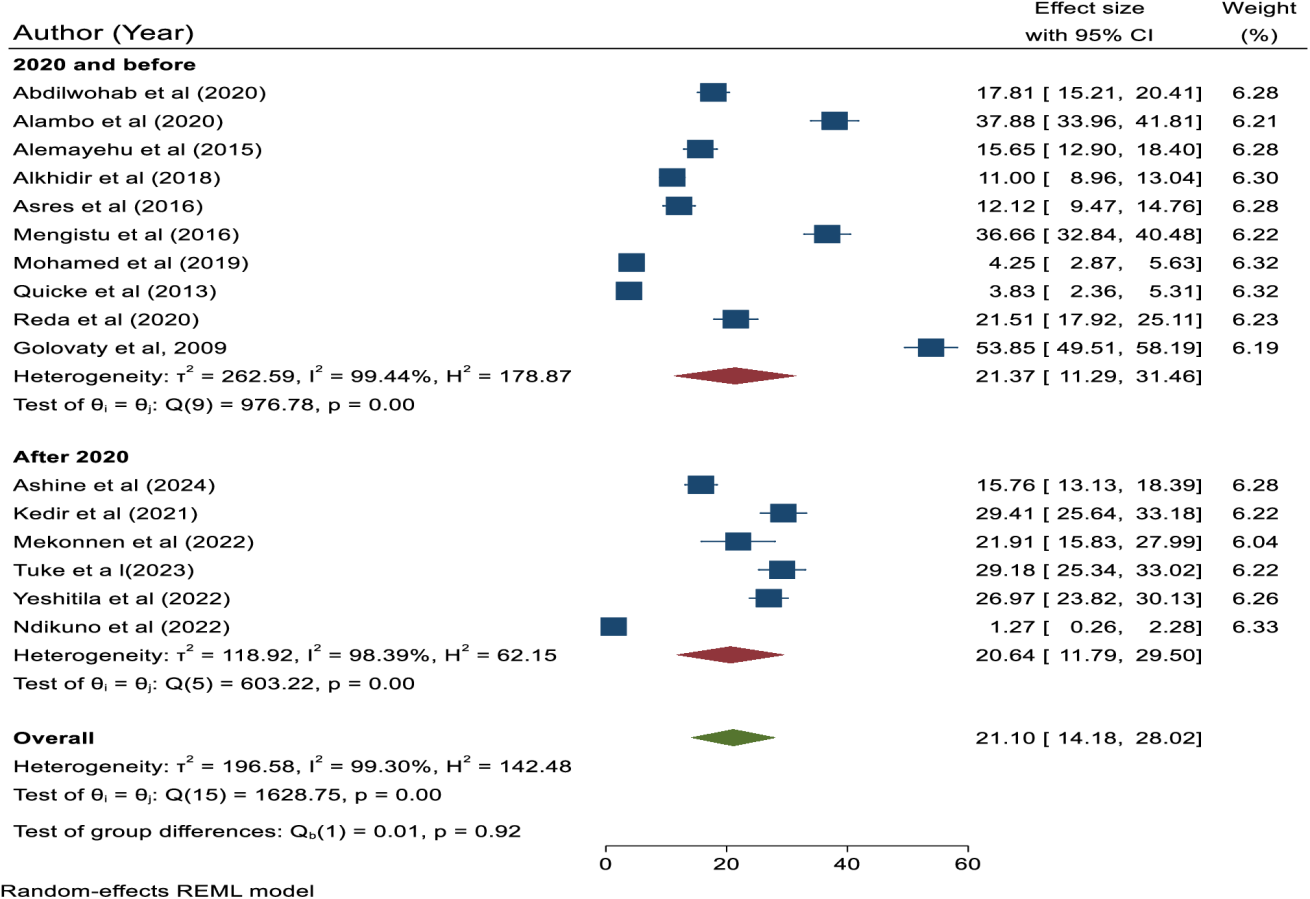
Subgroup analysis based on publication year category.

The subgroup analysis based on publication year category showed that the highest prevalence of active trachoma was reported in studies done in 2020 and before with a prevalence of 21.37% (95% CI: 11.29, 31.46), while the lowest prevalence was reported in studies published after 2020 where the prevalence of active trachoma was reported to be 20.64 (95%, CI: 11.79, 29.50). However, the pooled prevalence of active trachoma among studies done in 2020 and before and after 2020 did not show statistically significant differences (p = 0.92) (Fig 3**).**

**Fig 4:**
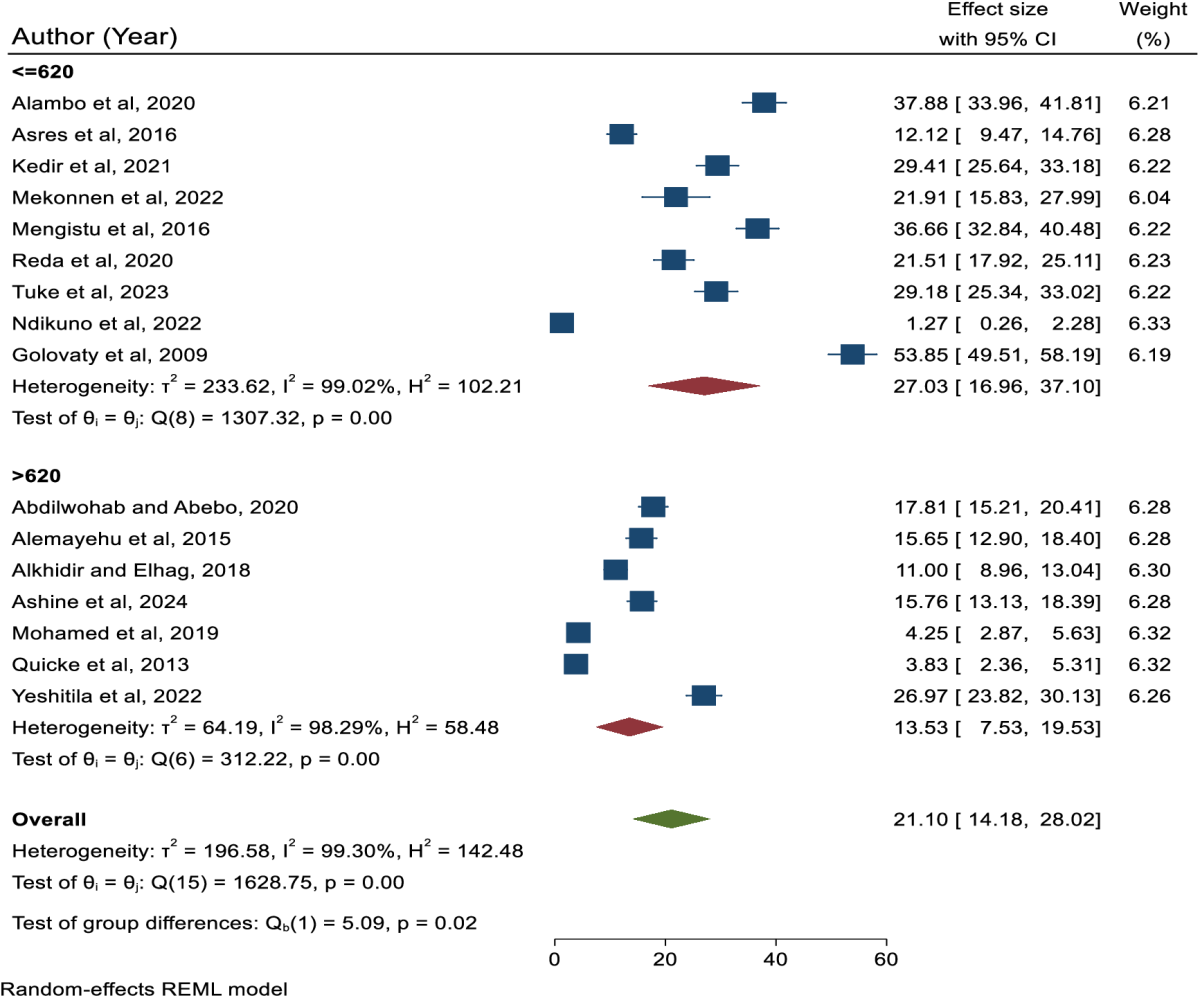
Subgroup analysis based on sample size.

The subgroup analysis based on the sample size category showed that the highest prevalence of active trachoma was reported in studies that contain a sample size of less than or equal to 620 with a prevalence of 27.03 (95% CI: 16.96, 37.10). The lowest prevalence was reported in studies containing a sample size of greater than 620 where the prevalence of active trachoma was reported to be 13.53% (95% CI: 7.53, 19.53), Moreover, the difference in the pooled prevalence of active trachoma among studies which contain sample size of less than or equal to 620 and greater than 620 was statistically significant.(p = 0.02) (Fig 4**).**

### Meta-analysis

#### Pooled prevalence of active trachoma.

**Fig 5:**
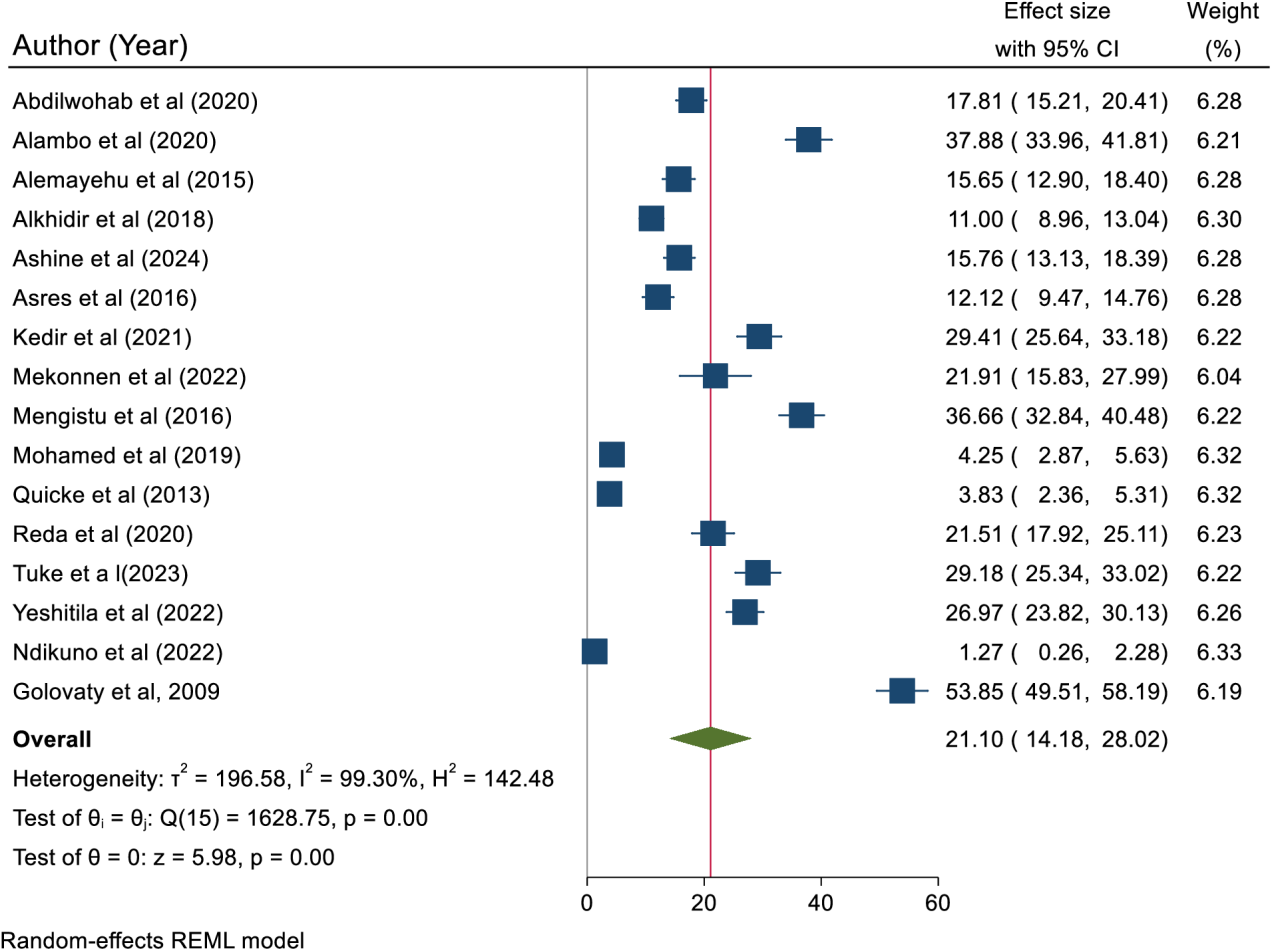
A forest plots showing the pooled prevalence of active trachoma among children 1-9 years old in low-income countries, 2024.

The inclusion of 16 articles revealed that the pooled prevalence of active trachoma among children aged 1–9 years was 21.10 (95% CI; 14.18, 28.02). The finding indicated that there was high heterogeneity among the included studies (I^2 ^= 99.3%, p < 0.0001). Due to this reason, a random effect model was used to estimate the pooled prevalence of active trachoma (Fig 5).

#### Publication bias assessment.

The potential publication bias was checked using statistical tests (Egger’s test) and visual inspection (funnel plot) method. The Egger’s test indicated that there is publication bias (p < 0.05) among studies included in this systematic review and meta-analysis (Table 2**).**

**Table 2:** Publication bias assessment using Egger’s test.

Study effect	Coefficient	Standard error	p-value	95% CI
Slope	−0.8357897	0.2643801	0.007*	−1.402829, −0.2687508
Bias	5.454584	0.5019598	<0.0001*	4.377988, 6.531181

P = 0.000 *Indicates the presence of statistical significance.

**Fig 6:**
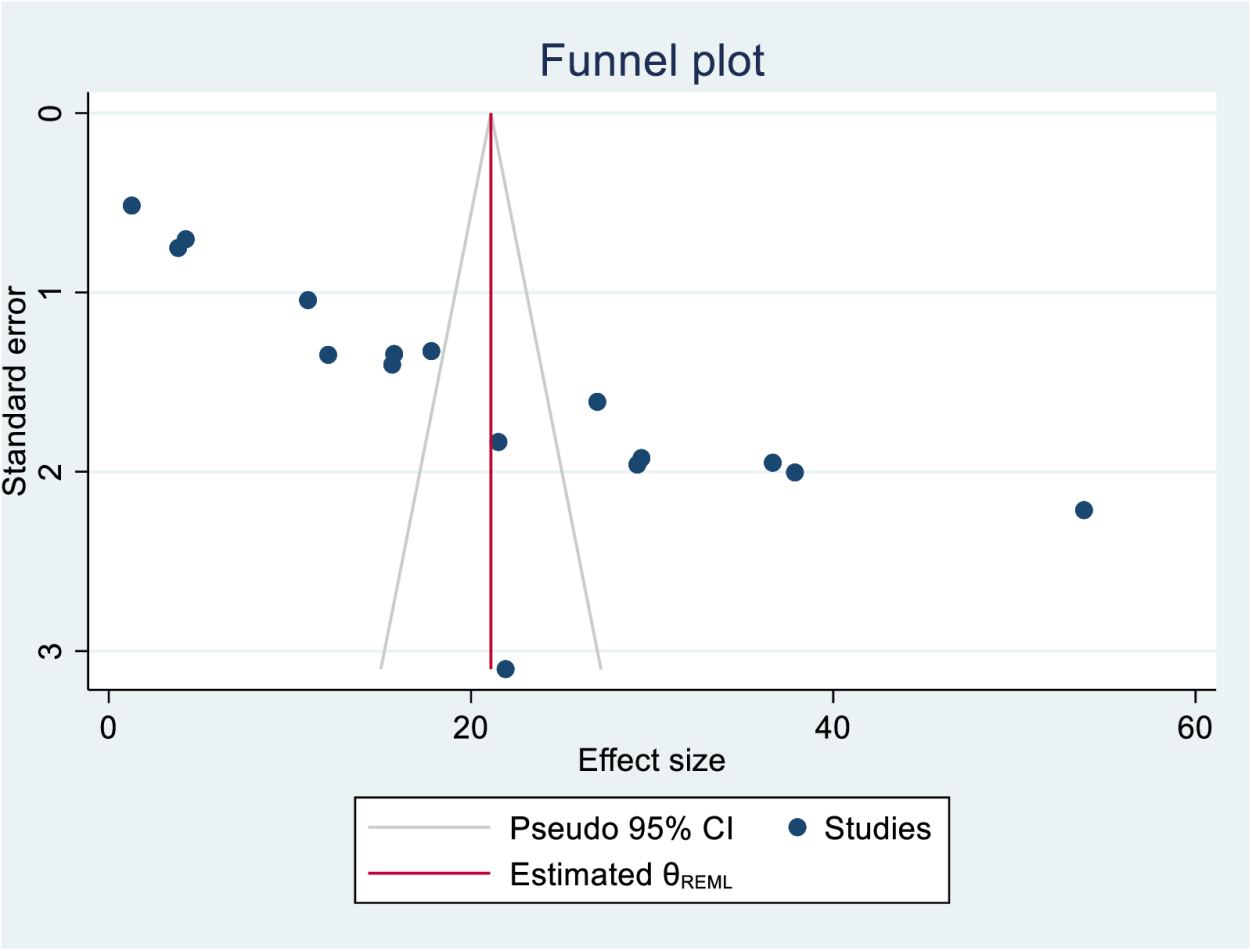
Funnel plot showing the presence of publication bias among studies included.

Similarly, from the funnel plot it is observed that the studies are not distributed symmetrically around the mean effect size, with smaller studies spread more widely at the bottom and larger studies clustered more closely at the top; this suggests the presence of publication bias (Fig 6**).**

**Fig 7:**
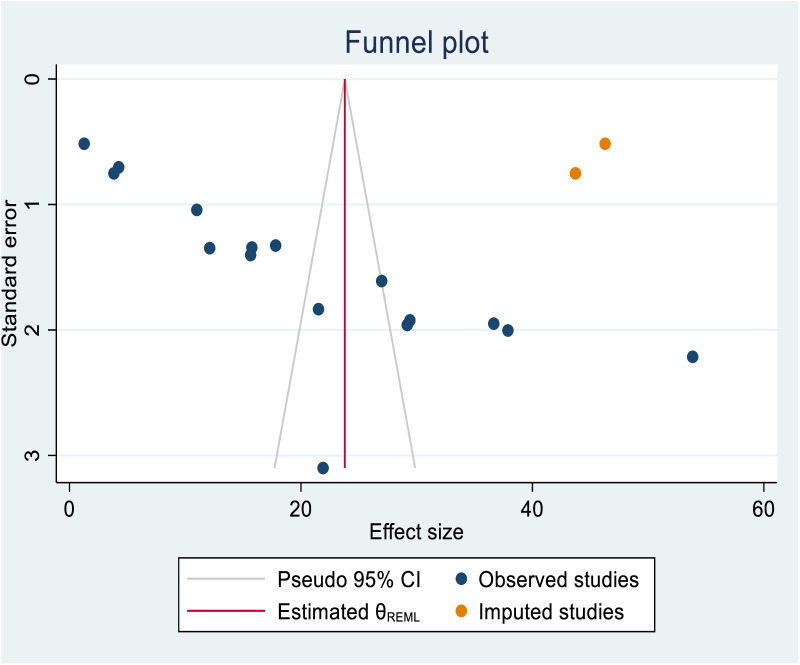
The funnel plot of a simulated meta-analysis after including two hypothetical studies to control the publication bias observed in this systematic review and meta-analysis study.

To determine the source of this bias, a trim and fill analysis was conducted. The finding indicates notable variation in the newly estimated pooled odds ratio, denoted as the adjusted point estimate (OR = 23.793; 95% CI: 16.685, 30.901) when compared to the initial or observed point estimate (OR = 21.1); 95% CI: 14.181, 28.020 (Fig 7**).**

### Sensitivity analysis

**Fig 8:**
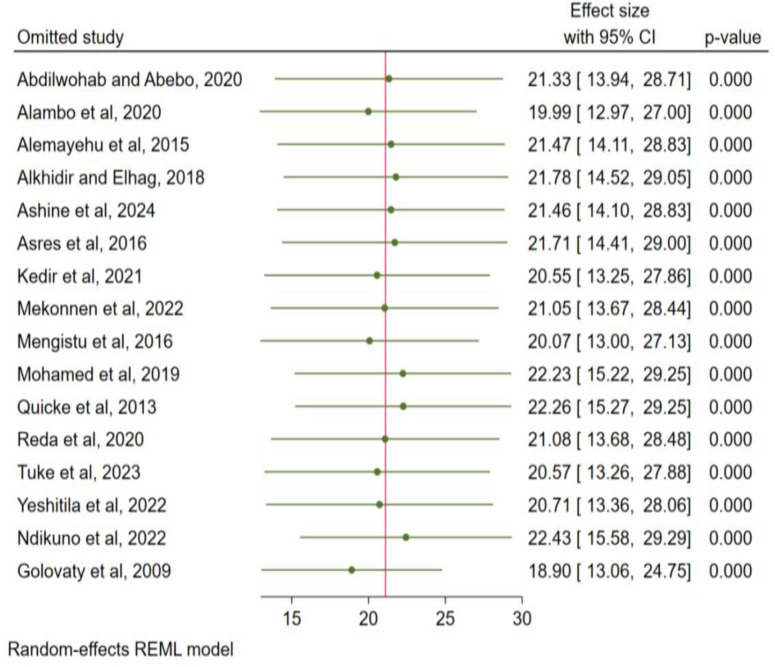
Sensitivity analyses for the systematic review and meta-analysis on the association between the presence of latrine and unclean child face and the pooled prevalence of active trachoma.

The impact of a single study effect on the pooled prevalence of active trachoma was checked using sensitivity analysis. As indicated below in the figure, when the first study [31] omitted from the model, the pooled prevalence of active trachoma was 21.33% (95% CI; 13.94, 28.71, p < 0.0001) and when the 2^nd^ study [32] removed from the model, the pooled prevalence of active trachoma was reported to be 19.99% (95% CI; 12.97, 27.00, p < 0.0001). When omitting and addition of a single study on the model continued, the pooled prevalence is in between the 95% confidence interval of the original pooled prevalence of active trachoma. Hence, there is no single study effect influencing the pooled prevalence of active trachoma (p < 0.001) (Fig 8**).**

### Meta-regression

To identify the potential sources of heterogeneity, meta-regression analysis was performed by considering the country category, publication year category, and sample size category as a factor. The finding indicates that the association between the study-level covariate i.e. country of the included study and the pooled prevalence of active trachoma among children aged 1 to 9 years in low-income countries is statistically significant (p < 0.05). This means that the observed relationship is unlikely to have occurred by chance alone, and there is evidence that the country of the included articles is a meaningful predictor of the pooled prevalence of active trachoma. The finding indicated that the source of heterogeneity is the country of the included studies. However, the publication year and sample size of the included studies did not significantly contribute to the occurrence of publication bias (Table 3**).**

**Table 3:** A univariate meta-regression analysis to identify the factors associated with the heterogeneity of studies included to determine the pooled prevalence of active trachoma among children aged 1 to 9 years in low-income countries, 2024.

Variables	Coefficient	Standard error	95% CI	p-value
Country category	16.48571	7.558378	1.671563, 31.29986	0.029*
Publication year category	−3.010403	6.071978	−14.91126, 8.890456	0.620
Sample size category	−11.03872	8.184984	−2.329231, 29.75532	0.068

*indicates a significant association.

### Association between unclean child face and the pooled prevalence of active trachoma

**Fig 9:**
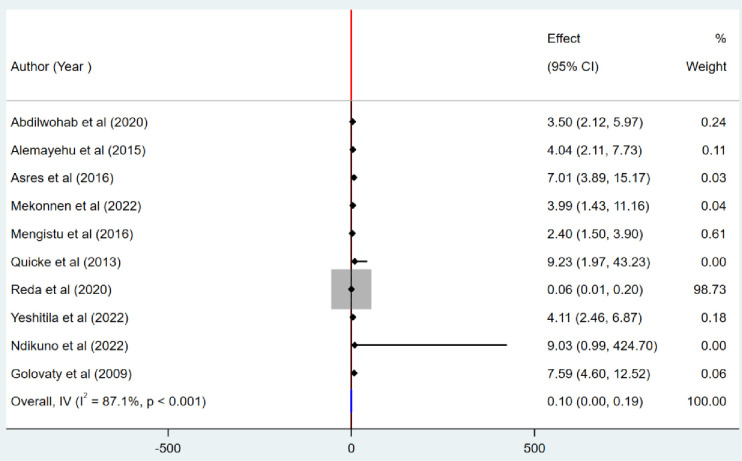
Association between unclean child face and the prevalence of active trachoma among children aged 1 to 9 years in low-income countries, 2024.

The presence of a statistical association between unclean child face and the pooled prevalence of active trachoma was studied by including ten articles [1,6,26,28,31,33–35,38,39]. The finding revealed that children who had unclean faces were 90% less likely to prevent the occurrence of active trachoma as compared to those who had clean faces (POR = 0.10, 95% CI; 0.00, 0.19) (Fig 9**).**

### Association between the presence of latrine and the pooled prevalence of active trachoma

**Fig 10:**
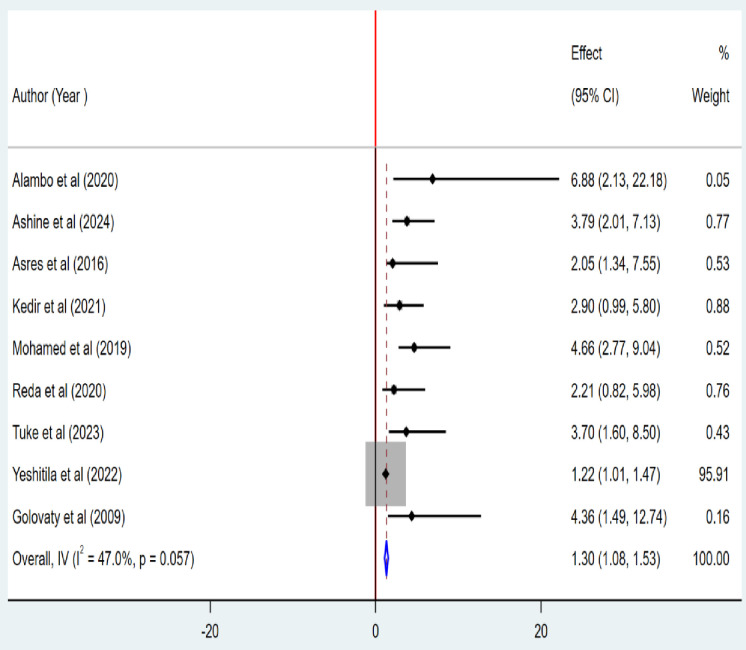
Association between the presence of latrine and the pooled prevalence of active trachoma among children aged 1 to 9 years in low-income countries, 2024.

The level of statistical association between the presence of latrine and the pooled prevalence of active trachoma was assessed using nine articles [1,25–29,32,36,40]. The finding indicated that there is a statistically significant association between the presence of latrines and the pooled prevalence of active trachoma among children aged 1 to 9 years in low-income countries. Those households who did not have a latrine facility were 1.3 times more likely to develop active trachoma than those who did not (POR = 1.30; 95% CI; 1.08, 1.53) (Fig 10**).**

## Discussion

The cornerstones of the WHO efforts to prevent trachoma, the primary infectious cause of blindness worldwide, are behavioral modification and environmental enhancements. Surgery, widespread antibiotic distribution, and the other elements of the SAFE strategy serve as the cornerstone for the eradication of blinding trachoma. Since Musca sorbens, the eye-seeking fly that contributes to the spread of the virus, primarily nests on human faces on the soil surface, several national initiatives have included efforts to construct home latrines as a way of vector management [14,41,42].

As with other Neglected Tropical Diseases (NTDs), trachoma typically affects rural areas with little resources. The majority of risk factors for trachoma are those that increase the likelihood of C. trachomatis spreading from person to person [16]. For this purpose, goal 3 of the Sustainable Development Goals calls for the eradication of NTDs including trachoma by 2030 [43]. It is one of the 20 NTDs, the most common infectious diseases in the world that cause blindness. It is the reason for the blindness or vision impairment of around 1.9 million of the world’s most impoverished individuals. Due to eyesight loss, trachoma results in a persistent cycle of poverty and poor health that makes it difficult for sufferers to work and take care of their families. It mostly affects the developing world’s most marginalized communities that lack basic amenities [19].

This systematic review and meta-analysis showed that owning a latrine has been linked to a lower chance of developing clinical indications of trachoma, according to risk factor analysis from multiple countries [41,44]. Owning a family latrine is independently linked to a decreased probability of exhibiting symptoms of active clinical trachoma, according to multivariate analyses of program evaluation data from different countries [45,46].

This study reported that the absence of a latrine facility increased the odds of active trachoma by 1.3 ((POR = 1.30; 95% CI; 1.08, 1.53). The lack of a latrine causes open defecation and favorably facilitates the reproduction of eye-seeking flies named Musca Sorbens, which explains the relationship between the absence of a latrine and greater odds of active trachoma. The most ideal and cozy medium for M. sorbens reproduction is human excrement that has been publicly defecated. Chlamydia trachomatis, the causative agent of active trachoma, is thereby spread to human faces by M. sorbens [14,19]. Research has demonstrated that fly control measures quadruple the risk of trachoma transmission [19]. According to a cluster-randomized controlled study examining the impact of latrines on the transmission of trachoma, the latrine intervention dramatically decreased the number of M. sorbens flies in the community and decreased the frequency with which flies landed in children’s eyes [41,47].

Numerous risk-factor analyses from Burkina Faso, Sudan, Egypt, and the Gambia have yielded a plethora of evidence indicating that the presence of a latrine in a family reduces the probability of an active case of trachoma [48–50]. The lack of a toilet in the complex was the most significant household factor linked to trachoma. Since residents of the community will inevitably urinate outside and contribute to an even higher fly population, this has consequences for environmental hygiene. Eliminating human faces from the environment will lower the population density since studies have indicated that isolated human faces on the soil surface serve as the ideal larval habitat for the trachoma vector, M. sorbens. It is imperative to prioritize environmental hygiene and the provision and usage of toilets as part of the control measures in these communities [51,52].

The presence of C. trachomatis in the environment and eye-seeking flies is more common in communities lacking access to bathrooms. This raises the possibility of transmission to people, particularly to small children who are particularly vulnerable to trachoma. The availability of latrines encourages sanitary practices like face and hand washing, which can lower the spread of trachoma. Other unsanitary behaviors that contribute to the disease’s spread may be linked to a lack of latrines. Enhancing the availability and utilization of sanitary amenities such as latrines is a crucial measure in the worldwide endeavor to manage and eradicate trachoma since it tackles various routes of disease propagation. The World Health Organization’s SAFE plan (Surgery, Antibiotics, Facial Cleanliness, and Environmental Improvement) for trachoma control includes improved sanitation as well as other WASH (Water, Sanitation, and Hygiene) measures [41,53].

According to this systematic review and meta-analysis study, children with dirty faces had a 90% lower chance of preventing the onset of active trachoma than those with clean faces (POR: 0.10, 95% CI: 0.19; 0.00). Other studies also reported similar findings [54,55]. This might be a result of the secretions’ capacity to draw in eye-seeking flies, which raises the possibility of trachoma spreading from person to person [47,56,57]. A dirty face can act as a reservoir for the bacteria and make it easier for the infection to spread to other people. Several studies have consistently demonstrated that having a dirty face is a significant risk factor for the development of active trachoma. Children who have a dirty face are more likely to get active trachoma than children who have a clean face. Children who have active trachoma, which is characterized by inflammatory signs, are also more likely to have increased eye and nasal discharge [52,54,58,59].

The primary individual trait linked to infection and trachoma was a dirty face [14,51]. As the frequency of face washing decreased and the children continued to have uncleaned faces free of discharge, the likelihood of active trachoma increased. The strongest proof of the connection between trachoma and hygiene parameters was also offered by other researchers. According to their research, there is a correlation between a lower risk of active trachoma and the following behaviors: cleaning your face more frequently, using soap, and keeping your face free of any discharge [9,17–21,23].

This review was based on a few articles, which may limit the statistical power and reliability of the meta-analysis findings. A larger number of studies from diverse low-income settings would be desirable to provide a more comprehensive assessment of the research question. Cross-sectional studies miss the dynamic changes in the variables over time, including the long-term effects of improvements in child face cleanliness or the availability of latrines on the prevalence of trachoma. To fully comprehend the historical link and changes in these characteristics, the inclusion of studies with a longitudinal or cohort design would be more appropriate. Socioeconomic status and hygiene practices that influence trachoma prevalence may not have been adequately controlled in the included studies. Additionally, the relevance of the findings could evolve over time as sanitation and public health initiatives improve, potentially limiting the applicability of older research.

Therefore, it is advised to broaden the review’s geographical scope to incorporate research from a greater variety of low-income contexts. In order to improve the meta-analysis’s statistical power and dependability, it is also recommended to incorporate a greater number of excellent researches. More original studies should also be performed to measure the prevalence and associated factors of active trachoma in all low-income countries. Furthermore, more longitudinal research ought to be carried out and incorporated into these kinds of systematic reviews and meta-analyses.

## Conclusion

The present systematic review and meta-analysis study revealed that the pooled prevalence of active trachoma was high compared to the WHO trachoma eradication goal which was passed in 2020. Active trachoma is still at the mesoendemic level in low-income countries. The presence of latrine facility and unclean child face were significantly associated with the prevalence of active trachoma among children aged 1 to 9 years in low-income countries.

Hence, Investing in the construction and promotion of latrine usage in affected communities would be an important intervention to reduce trachoma. In addition, implementing hygiene education programs, particularly targeting caregivers of young children, to emphasize the importance of regularly cleaning children’s faces could help lower trachoma rates. Since the pooled prevalence of active trachoma was found to be high compared to the WHO’s eradication goals, more concerted and comprehensive trachoma control measures are needed. This could include increased case detection, antibiotic distribution, and environmental improvements in the affected low-income country contexts.

## Supporting information

S1 Table PRISMA 2020 Checklist. (DOCX)

S2 Table JBI Quality Assessment Criteria. (DOCX)
